# Enzymatic Tenderization of Garden Snail Meat (*Cornu aspersum aspersum*) Using Plant Cysteine Proteases

**DOI:** 10.3390/molecules31030466

**Published:** 2026-01-29

**Authors:** Iwona Tesarowicz, Maciej Ligaszewski, Przemysław Pol, Krzysztof Surówka, Małgorzata Szczepanik, Katarzyna Widor, Karolina Budz

**Affiliations:** 1 Department of Biotechnology and General Technology of Food, Faculty of Food Technology, University of Agriculture in Krakow, Al. Mickiewicza 21, 31-120 Krakow, Poland; 2 National Research Institute of Animal Production, 1 Krakowska Street, Balice, 32-083 Krakow, Poland; 3 Independent Researcher, Ul. Balicka 122, 30-149 Krakow, Poland

**Keywords:** edible snail, *Cornu aspersum aspersum*, meat tenderization, papain, bromelain, ginger extract, cysteine protease

## Abstract

This study examined the impact of enzymatic tenderization on *Cornu aspersum aspersum* snail meat using mechanical texture analysis, SDS-PAGE (sodium dodecyl sulfate–polyacrylamide gel electrophoresis) protein profiling, and sensory evaluation. Samples were treated with papain (0.05–0.1%), bromelain (0.05–0.1%), or 10–25% ginger extract containing zingibain and subsequently hydrothermally processed. All enzymatic treatments significantly reduced shear force compared with the control (*p* < 0.05). The lowest connective tissue resistance (S1) was observed for 0.1% bromelain (14.1 N) and 10% ginger extract (19.0 N), versus >21 N in untreated samples. SDS-PAGE revealed that bromelain caused extensive degradation of high-molecular-weight proteins (>90 kDa), whereas papain induced moderate myofibrillar proteolysis. Higher concentrations of bromelain (0.1%) and ginger extract (25%) resulted in excessive softening and structural disintegration, leading to sensory disqualification. Sensory evaluation showed that moderate enzyme concentrations significantly improved overall acceptability (*p* < 0.05). Samples treated with 10% ginger extract achieved the highest overall sensory score (4.6/5), exceeding the control (3.9) and papain- or bromelain-treated samples (4.1–4.2). In conclusion, although bromelain exhibited the strongest proteolytic activity, a 10% ginger extract was identified as the optimal treatment, providing effective tenderization while preserving structural integrity and sensory quality.

## 1. Introduction

The global consumption of snail meat remains limited, as it is predominantly regarded as a luxury food product. Its regular consumption is largely confined to certain European countries, including France, Belgium, Italy, Spain, and Portugal, while it also plays a role in the culinary traditions of selected Asian and African regions. In recent years, however, increasing public interest in culinary diversification has been observed, accompanied by the growing popularity of exotic foods, among which snail-based dishes occupy a distinctive position [1,2,3].

Edible snail meat, particularly that of *Cornu aspersum aspersum*, represents a nutritionally valuable raw material, characterized by high protein content, essential vitamins, and mineral salts, while containing relatively low levels of fat. These attributes have led to its recognition as a component of healthy diets, frequently recommended by dietitians and nutritionists [1,2,4,5]. In addition to its favourable nutritional profile, snail meat is appreciated for its unique sensory properties. Nonetheless, the relatively high collagen content (approximately 1–3% [6,7,8,9,10]) poses significant technological challenges, as the meat requires prolonged hydrothermal treatment to achieve acceptable textural properties. Such processing is disadvantageous due to both organizational limitations in production and the degradation of heat-labile bioactive compounds. Moreover, the necessity of long cooking times and the resulting texture contribute to reduced consumer acceptance, despite the favourable nutritional profile of snail meat [11].

These limitations justify research efforts aimed at modifying and improving the texture of snail meat. In recent years, enzymatic approaches have attracted growing attention as tools for improving the texture and functionality of alternative protein sources, including non-traditional meats, mollusks, and insect-derived proteins [12,13,14,15,16,17]. It can therefore be assumed that the application of enzymatic tenderization methods may enhance the attractiveness of snail-based products, particularly in regions where there is no long-standing cultural tradition of their consumption. In the case of meat from warm-blooded animals, texture improvement is typically achieved through post-mortem enzymatic maturation or the use of exogenous proteolytic preparations. By analogy, the application of plant-derived proteolytic enzymes to snail meat represents a promising strategy to improve its technological and sensory properties [12,18,19].

The present study was designed to investigate the effect of selected cysteine proteases—papain (EC 3.4.22.2), bromelain (EC 3.4.22.4), and zingibain from ginger extract—on the structural properties of *C. aspersum* meat. Although these enzymes, widely studied in the context of meat tenderization, belong to the same family of cysteine proteases they differ in substrate specificity and cleavage preferences, resulting in distinct effects on myofibrillar and connective tissue proteins, particularly with regard to collagen degradation [12,20]. It was hypothesized that, despite the considerable collagen content, enzymatic treatment would promote partial hydrolysis of structural proteins, resulting in disruption of muscle tissue integrity. This, in turn, was expected to reduce the thermal processing time required to achieve a desirable texture, thereby improving the technological suitability and consumer acceptance of snail meat.

A review of the available literature indicates that research concerning edible snails has primarily focused on aspects related to farming, nutrition, and medicinal applications, while studies addressing their technological characteristics, particularly meat texture, remain relatively scarce [2,3,11,21]. Although enzymatic modification has been explored in other alternative protein matrices, its application to snail meat is still poorly documented. This knowledge gap underscores the novelty and scientific relevance of the present investigation. Furthermore, the potential practical outcomes of the study—namely, the identification of optimal enzymatic treatment conditions for the tenderization of snail meat—may contribute to the development of new, more widely acceptable snail-based products.

## 2. Results and Discussion

### 2.1. Texture Analysis Results

The texture of the analysed *C. aspersum* snail meat samples was evaluated using the Warner–Bratzler Shear Force (WBSF) test, an instrumental method commonly used in meat science to quantify mechanical properties such as tenderness and hardness [22]. The test involves measuring the maximum force required for a standardized blade to shear through the sample, providing objective data on the structural integrity of muscle and connective tissues.

In recent years, enzymatic tenderization has been increasingly explored not only in conventional meats, but also in alternative protein matrices such as mollusks and insect-derived muscle tissues. Studies on squid, shellfish and insect proteins have demonstrated that cysteine proteases may induce textural changes through selective degradation of myofibrillar and connective tissue proteins, although the extent and mechanism of these effects depend strongly on enzyme specificity and tissue architecture [12,20]. In this context, the application of plant-derived proteases to snail meat may be considered an extension of enzymatic strategies developed for structurally resistant, collagen-rich alternative protein sources.

In this study, the WBSF test was employed to assess textural differences between untreated samples and those subjected to various enzymatic tenderization protocols. According to Diakun [23,24], two primary forces are generated during the test: S1 (tendon shear force)—associated with resistance from connective tissues, representing hardness; and S2 (myofibrillar shear force)—associated with muscle fibres, representing tenderness. If S2 values are lower than S1, meat hardness is primarily associated with the resistance of connective tissue. Conversely, situations in which S2 approaches or exceeds S1 may reflect advanced enzymatic modification of muscle and connective tissue structures. However, such relationships should be interpreted with caution, as WBSF-derived S1 and S2 parameters provide indirect mechanical indicators of proteolysis rather than direct evidence of structural degradation [24].

Table 1 presents the WBSF results for *C. aspersum* snail meat, both untreated and after enzymatic and/or hydrothermal processing. In all cases, samples subjected to hydrothermal treatment demonstrated lower shear force values compared to untreated ones, confirming the tenderizing effect of cooking. This observation aligns with the established understanding that thermal processing denatures collagen, contributing to softer meat texture [25].

Across all treatment groups, the S1 values (related to connective tissue resistance) were consistently higher than S2 values (related to myofibrillar resistance), indicating persistent meat hardness primarily due to the rigidity of connective tissues. The lowest S1 value (14.1 N) was observed in samples treated with 0.1% bromelain combined with hydrothermal processing, suggesting this combination was most effective at breaking down collagen structures. Ginger extract also contributed to lowering S1 (to 19.0 N), although its effect was less pronounced than that of bromelain. The results support the hypothesis that proteolytic enzymes, particularly bromelain, improve meat tenderness when combined with hydrothermal treatment. This approach may also reduce cooking time and limit nutrient losses. Differences between some treatments were moderate.

Owing to organoleptic constraints, the 25% ginger extract was excluded from further analyses, with the exception of SDS-PAGE. Its use adversely affected sensory attributes, especially appearance, causing excessive degradation of the visceral sac and rendering it visually unappealing.

No prior studies have directly investigated the effect of enzymatic tenderization on snail meat texture. However, comparable findings from other meat types offer valuable insights. Buyukyavuz [26] assessed bromelain’s effects on raw duck breast using 1.5%, 3%, and 4.5% concentrations but found no significant differences in shear force among treated and control samples. In contrast, Kang and Rice [27] demonstrated that papain more effectively targets myofibrillar proteins, while bromelain preferentially hydrolyses collagen, suggesting complementary mechanisms of action. Gökoglu et al. [28] tested papain and bromelain on raw squid at 0.001% and 0.004% concentrations, reporting significant reductions in shear force. Calkins and Sullivan [29] documented a 20% reduction in beef shear force using just 0.009% papain—a statistically significant change—highlighting the importance of selecting enzyme type and concentration based on the specific meat matrix.

In the present study, bromelain (0.1%) was the most effective enzyme in reducing both S1 and S2 values, achieving nearly equal values for both forces after 1 h hydrothermal treatment. This reduction in S1 and S2 values indicates an advanced degree of enzymatic softening affecting both connective tissue and myofibrillar components. Nevertheless, WBSF parameters alone cannot be interpreted as direct evidence of complete structural breakdown and should be considered in conjunction with visual observations and SDS-PAGE results. However, despite the pronounced reduction in shear force, visual assessment revealed extensive softening and loss of structural integrity of the visceral sac. These macroscopic changes, together with the WBSF results, suggest excessive proteolytic activity rather than controlled tenderization.

In contrast, papain-treated samples retained relatively higher S1 values, indicating less effective collagen hydrolysis. These differences are consistent with mechanistic studies indicating that papain and bromelain, although both cysteine proteases, differ in substrate specificity and cleavage preferences. Papain has been shown to preferentially target myofibrillar proteins, whereas bromelain exhibits higher collagenolytic activity, resulting in more pronounced reductions in connective tissue-related shear force parameters. Such mechanistic divergence may explain the distinct S1 and S2 patterns observed in the present study [12,20,27,30].

Feng et al. [31] demonstrated that marination with 0.4% bromelain facilitated the production of fish balls with improved texture from intrinsically hard-textured fish (*Trachinotus blochii*). Similarly, enzymatic tenderization of squid (*Loligo vulgaris*) by proteolytic enzymes resulted in considerable softening of muscle tissue; in some cases papain produced superior textural effects compared with bromelain [28].

A broader comparative study at the University of Nebraska tested five GRAS-classified enzymes (Generally Recognized As Safe): papain, bromelain, ficin, bacterial, and fungal proteases. Among them, papain and bromelain produced the lowest shear force values in beef samples, with papain notably doubling the soluble collagen content. Interestingly, this increased solubility was attributed to thermal inactivation of all enzymes except papain at 70 °C [29], underlining the critical role of heat–enzyme interactions.

Singh et al. [32] reported that 2% bromelain reduced shear force by 44 N in chicken and 118 N in beef, confirming its effectiveness across species. Ketnawa and Rawdkuen [33] also showed that 20% bromelain caused rapid proteolysis of beef, chicken, and squid in just 1 h at room temperature—indicating that higher enzyme concentrations over short durations can replicate the tenderization effects of lower concentrations applied over longer periods.

However, extreme tenderization may compromise product integrity. For example, Melendo et al. [34] observed pronounced softening in squid, but also significant visual degradation—an outcome mirrored in the over-softened snail samples treated with 0.1% bromelain in this study.

Unlike papain and bromelain, which display broad substrate specificity, zingibain has been reported to preferentially hydrolyse collagen-rich structures while exerting milder effects on myofibrillar proteins, leading to more controlled tenderization [12,20]. Although zingibain (a protease from ginger) remains underexplored in snail meat tenderization, several studies confirm its strong proteolytic potential in different meat systems. Xiaozhen et al. [35] reported a significant reduction (*p* ≤ 0.01) in pork hardness using only 0.01% zingibain at 30 °C. Abdeldaiem and Hoda [36] further demonstrated that a 30% ginger aqueous extract effectively tenderized camel meat without compromising sensory quality. Moreover, when combined with gamma irradiation (1.5–4.5 kGy), the treatment extended refrigerated shelf life from 6 to 33 days.

In the present study, although 25% ginger extract improved the texture of the foot muscle, it caused excessive softening of the visceral sac, compromising the product’s sensory acceptability. Consequently, a 10% concentration was selected as the optimal balance between effective tenderization and maintaining product integrity.

Overall, the WBSF analysis demonstrated that enzymatic treatments effectively reduced mechanical resistance of snail meat, particularly in connective tissue-related parameters. Nevertheless, S1 and S2 values should be regarded as functional indicators of textural change rather than direct measures of protein degradation. Therefore, conclusions regarding the extent of proteolysis must be interpreted alongside electrophoretic and structural observations.

### 2.2. Sensory Evaluation of Enzymatically Tenderized Garden Snail Meat

The application of proteolytic enzymes markedly influenced the sensory properties of garden snail meat. As shown in Table 2, enzymatic treatment improved overall acceptability across most sensory attributes, with texture identified as the key factor driving consumer preference. Among all tested preparations, samples treated with 10% ginger extract achieved the highest overall sensory score (4.6), followed by those treated with 0.05% papain (4.2) and 0.05% bromelain (4.1). Each of these treatments significantly enhanced texture relative to the control (3.9), confirming that moderate enzymatic hydrolysis can effectively improve tenderness and palatability.

Samples treated with 0.1% bromelain solution and 25% ginger extract exhibited excessive tissue softening (overtenderization), particularly in the visceral sac region, resulting in structural disintegration. Due to unacceptable preliminary sensory characteristics, these samples were excluded from further sensory evaluation.

#### 2.2.1. Papain Treatments

Samples treated with 0.05% papain displayed substantial improvement in tenderness, reflected in both texture and overall sensory scores. However, a slight decline in appearance rating compared to the control was observed, likely due to partial collagen and myofibrillar protein hydrolysis that weakened surface integrity. Increasing the enzyme concentration to 0.1% resulted in over-tenderization, as evidenced by tissue softening and liquefaction in the visceral sac, which contributed to the lowest recorded overall score (3.3). These findings align with previous studies indicating that excessive proteolysis leads to undesirable texture and visual defects rather than sensory enhancement.

Mechanical testing using the Warner–Bratzler shear force method confirmed a reduction in cutting force with increasing papain concentration, indicating enhanced tenderness. However, no significant correlation was found (*p* > 0.05) between shear force parameters and sensory scores, suggesting that perceived texture depends not only on softness but also on the maintenance of structural cohesion. Similar results were reported by Istrati [37], who noted that while papain improves tenderness, excessive enzymatic activity may negatively affect mouthfeel without altering flavour perception. Consistent with these findings, the present study showed no significant effect of papain on flavour.

#### 2.2.2. Bromelain Treatments

The use of bromelain at a 0.05% concentration produced sensory scores comparable to those of papain-treated samples. The overall score of 4.1 and improved texture confirmed its efficacy as a natural tenderizer. However, the external appearance of bromelain-treated samples was slightly inferior to the control, probably due to advanced enzymatic hydrolysis of connective tissues in the visceral sac region. Increasing the bromelain concentration to 0.1% led to complete disintegration of the tissue, and these samples were consequently disqualified from visual assessment. These results corroborate the findings of Saengsuk [38], who reported similar concentration-dependent effects of bromelain on pork steaks.

Aroma and flavour ratings of bromelain-treated samples remained within the control range, with only slight aromatic enhancement observed at higher enzyme levels. These results confirm that bromelain primarily affects textural attributes without substantially altering other sensory parameters.

#### 2.2.3. Ginger Extract Treatments

The 10% ginger extract treatment demonstrated the most favourable sensory profile, with superior texture, natural appearance, and pleasant aroma. Samples treated with this concentration exhibited pronounced tenderness improvement (texture score 1.4) without signs of tissue degradation. In contrast, the 25% extract led to excessive proteolysis and visual disqualification. The mild yet effective proteolytic activity of zingibain from ginger extract likely contributes to its balanced performance, tenderizing internal structures while preserving surface integrity.

These findings are consistent with previous research by Naveena and Mendiratta [39] and Abdeldaiem and Hoda [36], who reported improved juiciness, colour, and overall acceptability in poultry and camel meat following enzymatic treatment with ginger extract. The present results extend these observations to garden snail meat, indicating that zingibain contained in ginger can serve as an efficient and consumer-acceptable tenderizer for non-traditional protein sources.

#### 2.2.4. Overall Interpretation

Taken together, the results demonstrate that controlled enzymatic tenderization is an effective method for improving the sensory quality of snail meat. As shown in Table 3, moderate enzyme concentrations, particularly 0.05% papain, 0.05% bromelain, and 10% ginger extract, enhanced tenderness and overall acceptability without compromising appearance or flavour. However, excessive enzyme levels led to structural degradation, underscoring the importance of optimizing treatment conditions. Among the tested enzymes, zingibain contained in ginger extract exhibited the most balanced sensory performance, improving texture and consumer appeal while maintaining product integrity—making it the most promising candidate for industrial or culinary applications in snail meat processing.

### 2.3. SDS-PAGE Electrophoresis Results

**Figure 1:**
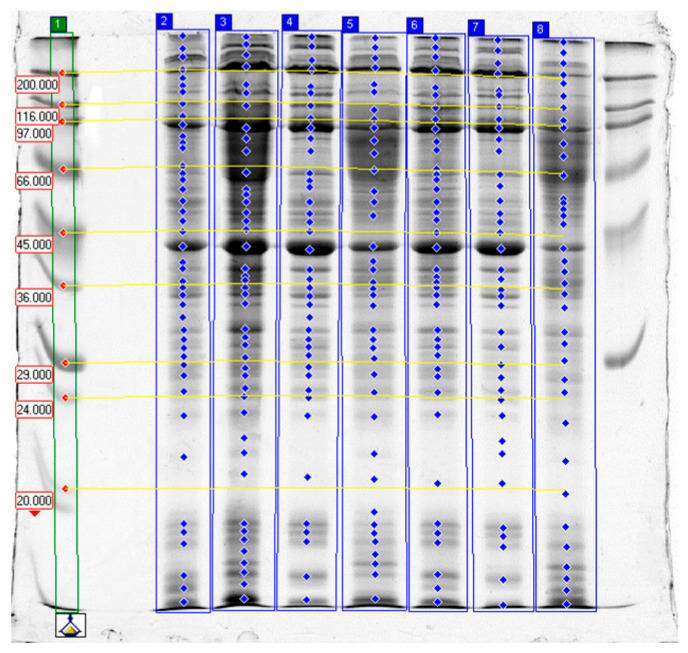
The results of SDS-PAGE of garden snail meat tenderized with papain. Lanes: 1—high- and low-molecular-weight standards (Da); 2–5—blind samples: whole carcass (2), whole carcass (double volume) (3), foot (4), visceral sac (5); 6–8—samples incubated in buffer: whole carcass (6), foot (7), visceral sac (8); the last, unmarked line—high-molecular-weight standards.

**Figure 2:**
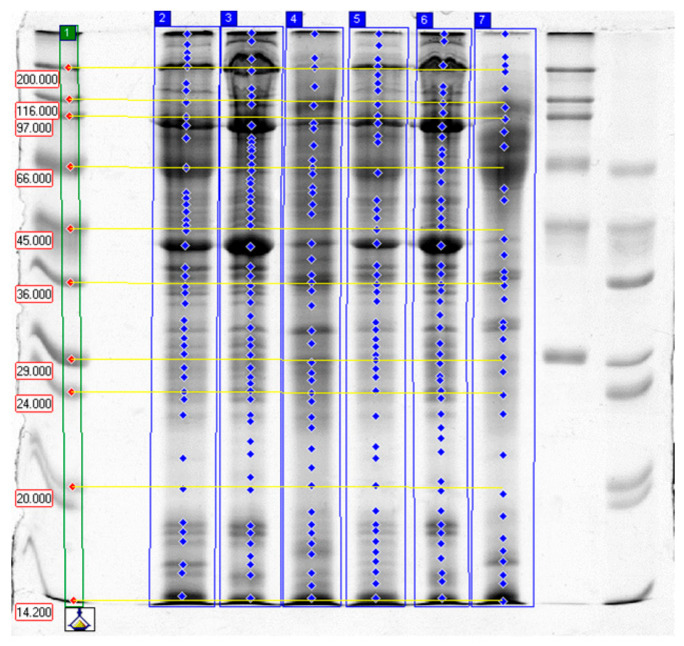
The results of SDS-PAGE of garden snail meat tenderized with papain. Lanes: 1—high- and low-molecular-weight standards (Da); 2–4—samples incubated in 0.05% papain: whole carcass (2), foot (3), visceral sac (4); 5–7—samples incubated in 0.1% papain: whole carcass (5), foot (6), visceral sac (7); the last two, unmarked lines-high and low molecular-weight standards.

**Figure 3:**
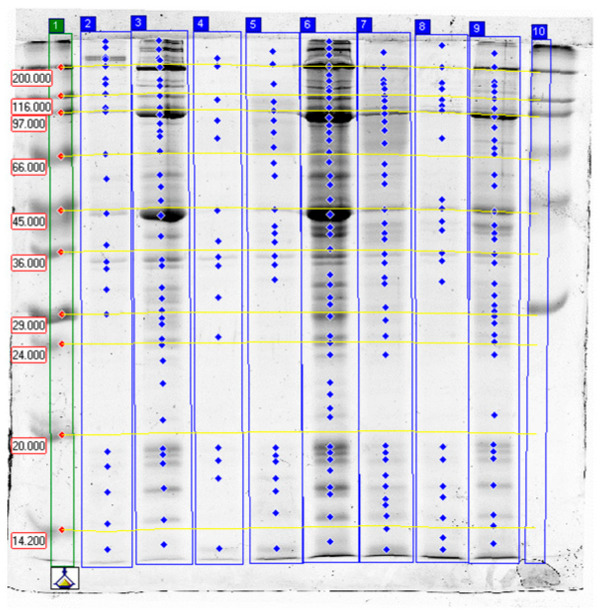
The results of SDS-PAGE of garden snail meat tenderized with ginger extract. Lanes: 1—high- and low-molecular-weight standards (Da); 2–4—blind samples: whole carcass (2), foot (3), visceral sac (4); 5–7—samples incubated in 10% ginger extract: whole carcass (5), foot (6), visceral sac (7); 8–9—samples incubated in 25% ginger extract: whole carcass (8), foot (9); 10—high-molecular-weight standards.

Results of SDS-PAGE are presented in Table 4 and Table 5 as well as on Figure 1, Figure 2 and Figure 3, which show selected separations. The analysis of band intensities was based on single separations and was of indicative nature.

Electrophoretic SDS-PAGE analysis of proteins from garden snail (*C. aspersum*) muscle confirmed that the proteolytic activity of papain, bromelain, and ginger extract varied depending on the enzyme type, its concentration, and the tissue subjected to treatment (whole carcass vs. foot muscle only). In all control samples, high-molecular-weight fractions predominated, corresponding to intact structural proteins—myosin heavy chains (>200 kDa), actin (~42 kDa), tropomyosin (34–39 kDa), and paramyosin—which is characteristic of molluscan muscles (~95–115 kDa) [40,41,42,43,44].

#### 2.3.1. Effects of Papain

Papain exhibited moderate proteolytic activity. Increasing the enzyme concentration from 0.05% to 0.1% resulted only in a slight decrease in high-molecular-weight fractions and a moderate rise in lower-molecular-weight fragments (e.g., 50–90 kDa), indicating partial myosin hydrolysis [41]. These changes were more pronounced in whole-carcass samples than in the foot, which may be attributed to differences in tissue architecture and greater enzyme accessibility in softer tissues.

Papain is known for its controlled activity toward myofibrillar proteins and moderate hydrolysis rate. The absence of a strong increase in low-molecular-weight fragments (<40 kDa) confirms the mild character of the proteolysis, consistent with previous reports [12,13,45,46].

The literature indicates that papain activity strongly depends on experimental conditions (pH, temperature, enzyme–substrate ratio) as well as the form of the enzyme preparation. In practice, papain effectively degrades myofibrillar proteins and, in some cases, connective-tissue proteins; however, its efficiency and optimal concentration range vary considerably across studies [12,13,20,45]. This may explain the more moderate effect of papain in the present study compared with bromelain.

#### 2.3.2. Effects of Bromelain

The most pronounced proteolytic effect was observed in samples treated with bromelain. Particularly at the higher concentration (0.1%), a clear reduction in the intensity of fractions >90 kDa and a marked increase in fragments within the 60–70 kDa range and below 30 kDa were detected. This pattern indicates extensive hydrolysis of myosin and paramyosin into smaller fragments [31,43,44].

Similar findings were reported by Ketnawa and Rawdkuen [33], who demonstrated strong bromelain-induced hydrolysis of myosin heavy chains and actin in poultry and beef, as well as paramyosin in squid muscle. The high activity of this enzyme is attributed to its broad substrate specificity and ability to hydrolyse both myofibrillar proteins and collagen [12,13,33,47]. Such extensive proteolytic capacity makes bromelain an effective tenderizing agent; however, excessive enzyme concentration or prolonged exposure may lead to over-tenderization, characterized by excessive protein breakdown and loss of structural integrity.

The proteolytic effect of bromelain was particularly pronounced in whole-carcass samples, which exhibited highly diversified medium- and low-molecular-weight fractions indicative of advanced protein hydrolysis. In contrast, the process was somewhat less intense in foot muscle samples, likely due to limited enzyme penetration into the more compact tissue structure. A similar observation was noted by Bekhit [13].

#### 2.3.3. Effects of Ginger Extract

Ginger extract demonstrated effective but variable proteolytic activity: in several cases, the 10% extract caused a noticeable increase in lower-molecular-weight fractions (e.g., 68–120 kDa), whereas raising the concentration to 25% did not always enhance this effect. The literature confirms that zingibain, ginger protease, exhibits lower activity toward myofibrillar proteins compared with bromelain and papain, while efficiently hydrolyzing collagen, making it potentially useful for controlled meat tenderization [12,13,20,36,48,49,50].

The present results confirm that zingibain contained in ginger extract acts in a selective and controlled manner, producing limited proteolysis of myofibrillar proteins without causing rapid disruption of muscle structure. Abdeldaiem and Hoda [36] similarly reported that ginger extract induces gradual hydrolysis of myosin and collagen, improving camel meat tenderness while maintaining structural cohesion. The moderate proteolytic intensity observed in this study may reflect the enzyme’s higher affinity for collagen and connective-tissue proteins than for typical myofibrillar components.

In foot muscle samples, a moderate decrease in high-molecular-weight fractions and the appearance of additional bands around 90–100 kDa were noted, likely corresponding to partial hydrolysis of myosin heavy chains into lower-molecular-weight segments [41]. In whole-carcass samples, the effect was more pronounced but still weaker than that produced by bromelain.

The literature describes zingibain as having strong collagenolytic activity and often exhibiting activity optima distinct from those of bromelain and papain. Differences in enzyme form (raw extract vs. purified enzyme) and reaction conditions can therefore lead to non-linear concentration–response patterns, consistent with the irregularities observed between the 10% and 25% treatments [12,13,20,48,49,50,51].

#### 2.3.4. Effect of Tissue Type

Comparison of the “whole carcass” and “foot” samples indicates that tissue characteristics substantially affect enzymatic hydrolysis efficiency. Whole carcasses contain looser muscle structures and a higher proportion of soluble proteins, facilitating enzyme access to substrates. In contrast, the compact fiber arrangement in the snail foot provides a more resistant matrix for enzymatic action. These findings demonstrate that the extent of proteolysis depends not only on enzyme type but also on the physical properties of the tissue, consistent with earlier reports [33].

#### 2.3.5. Summary and Practical Implications

From a practical standpoint, controlling the degree of proteolysis is crucial, as uncontrolled protease activity leads to over-tenderization—characterized by the formation of short, soluble peptides and the loss of desirable texture—an issue repeatedly highlighted in review studies. Therefore, both literature and the present results argue for kinetic (time-course) analyses, extended concentration ranges, standardized protein loading, and the identification of peptide fragments to confirm which proteins undergo hydrolysis.

Overall, the findings confirm that bromelain is the most effective protease under the experimental conditions applied. Papain exhibits moderate activity, whereas ginger extract shows variable proteolytic effects depending on concentration and tissue type. These results underscore the importance of selecting an appropriate enzyme and concentration to achieve the desired degree of proteolysis in muscle tissues. The outcomes align with published data indicating that bromelain possesses broad specificity and strong capacity to hydrolyze myosin and collagen, while papain and zingibain show more controlled activity [12,13,33,36,52].

## 3. Materials and Methods

### 3.1. Raw Material

Garden snails (*C. aspersum*) in the amount of 928 were obtained from the National Research Institute of Animal Production in Balice (Krakow, Poland). Specimens were cooled at 2 °C for 1.5 h (Whirlpool refrigerator AND 480S, Whirlpool EMEA S.P.A., Milano, Italy) and subsequently frozen by shock freezing at −40 °C for 1 h (shock freezer ZLN-T 300 (-70), COCH, Krakow, Poland). After that, the specimens were immersed in boiling water and maintained at 100 °C for 3 min. This thermal treatment facilitated the detachment of the soft tissues from the shells without causing significant thermal denaturation. Soft tissues were manually separated from the shells and rinsed with distilled water, subsequently gently dried using a paper towel. The foot regions of the carcasses were pierced at five locations to facilitate enzyme penetration, while the visceral sacs were left intact due to their delicate structure. All carcasses were divided into 8 groups, two of which were designated as control/blank groups, and the remaining ones were designated for incubation in appropriate enzyme solutions.

### 3.2. Enzyme Preparations

Papain and bromelain, both with labelled activities of 3 U/mg, were obtained as commercial reagents (Sigma-Aldrich, St. Louis, MO, USA) and prepared as citrate buffer solutions (pH 6.0) at *w*/*w* concentrations of 0.05% and 0.1%. Zingibain was applied as an extract of ginger rhizomes, prepared by homogenizing peeled rhizomes with distilled water in a 1:1 ratio, followed by filtration through gauze. The extract was subsequently diluted to *w*/*w* concentrations of 10% and 25%.

### 3.3. Sample Treatment

Carcasses were immersed in the respective enzyme solutions and incubated at 4 °C for 24 h (Table 6). Following incubation, samples were removed from the refrigerator and rinsed with distilled water. For each treatment variant, the samples were divided into two groups. One group was subjected to hydrothermal treatment in a vegetable broth, prepared from 2 L of water, 150 g of carrot, 100 g of zucchini, 120 g of parsley, 2 cloves of garlic, 100 g of celery, 50 g of onion, 2 bay leaves, 1 teaspoon of salt, a quarter teaspoon of pepper, 1 teaspoon of herbs de Provence, and a stock cube, and cooked for 1 h. The second group was briefly boiled for 5 min in 2 L of water solely to inactivate the enzyme and subsequently used directly for analysis.

For ginger extract, only samples incubated in the 10% extract were included in the textural and sensory analysis, as preliminary organoleptic evaluation indicated that the 25% extract was excessively concentrated and negatively affected the sensory properties of the carcasses. Samples treated with 0.1% bromelain solution were excluded from sensory studies for the same reason.

### 3.4. Control Treatments

Parallel blind trials were performed, in which the samples were incubated either in citrate buffer (pH 6.0) or in distilled water (for comparison with ginger extract-treated samples). Following incubation, all control samples were subjected to the same cooking conditions as those applied to the enzyme-treated counterparts.

### 3.5. Texture Analysis

Textural properties of the samples were measured using the Warner–Bratzler Shear Force method [53]. Maximum cutting force, indicative of meat tenderness, was determined with a TA-XT2 Texture Analyser (Stable Micro Systems Ltd., Godalming, Surrey, UK). Prepared carcasses were positioned on the table so that the knife, equipped with a triangular blade, cut both the foot and visceral sac portions in the same orientation. The knife advanced at a rate of 1 mm/s. Measurements were performed tenfold for each variant group (after thermal treatment and without) with the use of a separate snail carcass for each cut.

### 3.6. Sensory Evaluation

The sensory evaluation of garden snail meat (appearance, taste, colour, aroma, and texture) was performed by a trained panel of 15 assessors (9 women and 6 men), aged 25–49 years. Panelists were recruited in accordance with ISO 8586:2012 [54], with both health and sensory criteria considered. All assessors were in good health, reported no food allergies or sensory impairments, and none were smokers or taking medications that could influence sensory perception.

The evaluations were carried out in a controlled sensory laboratory, under neutral lighting and temperature conditions, and free from extraneous odours. Each sample containing 3 carcasses of a given variant was served in coded plastic cups at 21 ± 1 °C, with room-temperature water provided for palate cleansing between assessments. Sensory attributes were evaluated using a five-point descriptive scale. To ensure unbiased sensory assessment and minimize order and carry-over effects, the presentation order of samples was randomized for each assessor and each evaluation session. A balanced randomization scheme was applied so that all treatment variants appeared with similar frequency in each serving position across panelists. Samples were identified using randomly generated three-digit numerical codes, which were reassigned between sessions. The assessors were blinded to sample identity and treatment type throughout the evaluation. Each assessor evaluated all samples in a randomized order during two independent sessions conducted on separate days. The detailed evaluation criteria are presented in Table 7.

### 3.7. SDS-PAGE Electrophoresis

Protein profiles were analysed separately for the foot muscle and the whole soft body of the snails. All untreated, blind and enzyme-treated samples, consisting of 3 carcasses for each variant, were homogenized in distilled water to obtain suspensions with an approximate protein concentration of 1%. Aliquots of the homogenates were mixed at a 1:1 ratio with a denaturing buffer containing 0.125 M Tris (pH 6.8), 4% SDS, 20% glycerol, and 2% 2-mercaptoethanol. All reagents were purchased from Sigma-Aldrich (St. Louis, MO, USA). The mixtures were then heated in a boiling water bath for 90 s to denature the proteins, followed by centrifugation at 5000× *g* for 15 min (Centrifuge MPW-56, MPW Med. Instruments, Warszawa, Poland). The resulting supernatants were used for electrophoretic analysis.

Sodium dodecyl sulphate-polyacrylamide gel electrophoresis (SDS-PAGE) was performed according to the Laemmli [55] protocol, using gels with a final acrylamide concentration of 12.5% (*w*/*v*). Samples were loaded in the amount of 5 µL and protein molecular weights were estimated based on reference standards MW-SDS-200 and MW-SDS-70 L (Sigma-Aldrich, St. Louis, MO, USA). Electrophoresis was carried out using a Hoefer Mighty Small II SE 260 vertical system coupled with an EPS 301 power supply unit (Hoefer Inc., Bridgewater, MA, USA). The separation was conducted at a constant current of 40 mA and lasted approximately 85 min until the electrophoresis front reached 5 mm to the end of the gel.

For protein visualization, gels were stained with 0.025% Coomassie Brilliant Blue R-250 in a solution of 40% methanol and 7% acetic acid for 1 h. Destaining was conducted using the same methanol/acetic acid mixture, which was replaced periodically until a clear gel background was achieved. Electrophoretic patterns were analysed using Image Master TotalLab v. 1.0 software (Amersham Pharmacia Biotech, Uppsala, Sweden) to obtain qualitative and semi-quantitative assessments of the samples. The received band intensities and proportions are only estimates.

### 3.8. Statistical Analysis

Results were reported as mean values with corresponding standard deviations (n = 10 in WBSF measurements and n = 15 in sensory analysis). Statistical evaluation was carried out using Statistica version 14.0 (StatSoft, Krakow, Poland). Group comparisons were performed using one-way ANOVA with Tukey’s post hoc test. Statistical significance was defined as *p* < 0.05.

## 4. Conclusions

Electrophoretic SDS-PAGE and texture analyses demonstrated that the effectiveness of the tested proteases—papain, bromelain and zingibain contained in ginger extract—in modifying the properties of garden snail (*C. aspersum*) meat depended on enzyme type, concentration and the tissue subjected to treatment (whole carcass vs. foot). Bromelain exhibited the highest proteolytic activity, substantially degrading both myofibrillar proteins and collagen; this was reflected by low Warner–Bratzler shear force values and a marked reduction in high-molecular-weight fractions in SDS-PAGE. However, its strong activity carried a risk of excessive structural degradation, which in extreme cases reduced sensory acceptability.

Papain was characterized by moderate and more controlled proteolysis, producing a notable but not excessive softening of the meat. Its effect was particularly evident in whole-carcass samples, where enzyme access to substrates was greater than in the compact foot tissue. Ginger extract showed selective activity—effectively hydrolyzing connective-tissue proteins while producing limited hydrolysis of myofibrillar proteins. The most favourable sensory properties were obtained with 10% ginger extract; higher concentration (25%) caused undesirable degradation of visceral sac tissues.

Tissue type significantly influenced the course of proteolysis: the looser structure of whole carcasses promoted enzyme action, whereas the compact foot muscle was more resistant to hydrolysis. Overall, these results confirm that appropriate selection of enzyme and concentration is critical to achieve the desired level of tenderness without compromising product integrity.

In summary, bromelain is the most potent tenderizing enzyme but requires precise control to avoid over-degradation of tissue structure; papain acts more mildly and can be used when gradual softening with preserved tissue cohesion is desired; and ginger extract provides the most balanced sensory outcome, improving texture while maintaining visual and structural integrity, and thus appears most promising for culinary and industrial applications.

These results highlight the need for further kinetic and structural studies across broader concentration ranges and exposure times and with precise identification of generated protein fragments to optimize enzymatic tenderization protocols for snail meat and other muscle substrates.

Studies using ginger extract at concentrations ranging from 5% to 15% seem particularly worthwhile, as does research examining the activity of the pure zingibain. Investigating the effect of temperature on the tenderizing effects of the enzymes studied in this work also seems crucial. The methodology should certainly be expanded to characterize the final effects of proteolysis.

## Figures and Tables

**Table 1:** Results of WBSF texture analysis.

		Samples Without 1 h Hydrothermal Treatment		Samples with 1 h Hydrothermal Treatment (HT)	
		S1 [N]	S2 [N]	S1 [N]	S2 [N]
blind test *	for ginger extract	28.2 ± 1.6 ^a^	25.2 ± 1.9 ^a^	21.4 ± 0.7 ^a^	20.9 ± 1.0 ^a^
	for papain and bromelain	30.3 ± 3.3 ^a^	24.3 ± 2.3 ^a^	22.7 ± 4.3 ^a^	21.8 ± 3.6 ^a^
papain 0.05%		24.8 ± 1.3 ^b^	20.1 ± 2.2 ^a^	18.3 ± 0.8 ^ab^	17.4 ± 0.3 ^b^
papain 0.1%		22.9 ± 1.2 ^b^	17.2 ± 0.5 ^b^	18.0 ± 0.9 ^ab^	15.1 ± 1.1 ^c^
bromelain 0.05%		18.2 ± 3.3 ^bc^	15.0 ± 4.7 ^bc^	17.6 ± 3.2 ^b^	15.4 ± 3.8 ^bc^
bromelain 0.1%		14.2 ± 3.3 ^c^	12.7 ± 2.7 ^bc^	14.1 ± 3.7 ^b^	14.1 ± 3.6 ^bc^
ginger extract 10%		26.0 ± 1.6 ^ab^	20.0 ± 2.1 ^ab^	19.0 ± 1.9 ^ab^	18.9 ± 1.7 ^b^

S1—tendon shear force (hardness); S2—myofibril shear force (tenderness). The presented values are means ± standard deviations (n = 10). Values with different superscripts (a–c) in columns differ significantly at *p* ≤ 0.05. * The treatment procedures for blind tests are presented in the “Control Treatments” Section in Materials and Methods part.

**Table 2:** Results of sensory evaluation of snail meat samples.

		Appearance	Colour	Aroma	Taste	Texture	Final Score
Weight factor		0.20	0.15	0.15	0.20	0.30	
Blind test	average rating	4.3 ± 0.6 ^a^	3.8 ± 0.6 ^a^	4.3 ± 0.5 ^a^	3.7 ± 0.6 ^b^	3.7 ± 0.6 ^ab^	
	score	0.9	0.6	0.7	0.7	1.0	3.9 ± 0.3 ^ab^
Papain 0.05%	average rating	4.1 ± 0.6 ^a^	3.9 ± 0.6 ^a^	4.6 ± 0.5 ^a^	4.0 ± 0.8 ^ab^	4.3 ± 0.6 ^ab^	
	score	0.8	0.6	0.7	0.8	1.3	4.2 ± 0.4 ^a^
Papain 0.1%	average rating	2.2 ± 0.4 ^b^	3.5 ± 0.6 ^a^	4.0 ± 0.7 ^a^	4.1 ± 0.7 ^ab^	3.1 ± 0.6 ^b^	
	score	0.5	0.5	0.6	0.8	1.0	3.3 ± 0.4 ^b^
Bromelain 0.05%	average rating	3.9 ± 0.5 ^a^	3.6 ± 0.6 ^a^	4.3 ± 0.5 ^a^	4.5 ± 0.6 ^ab^	4.3 ± 0.5 ^ab^	
	score	0.8	0.5	0.6	0.9	1.3	4.1 ± 0.3 ^a^
Bromelain 0.1%	disqualifying visual assessment						
Ginger extract 10%	average rating	4.2 ± 0.4 ^a^	4.5 ± 0.5 ^a^	4.8 ± 0.4 ^a^	4.8 ± 0.4 ^a^	4.9 ± 0.4 ^a^	
	score	0.8	0.7	0.7	1.0	1.4	4.6 ± 0.2 ^a^
Ginger extract 25%	disqualifying visual assessment						

The presented values are means ± standard deviations (n = 15). Values with different superscripts (a, b) in columns differ significantly at *p* ≤ 0.05.

**Table 3:** Comparative summary of enzymatic effects on sensory attributes of snail meat. The symbol ↑ indicates improved properties.

Enzyme	Concentration	Texture Improvement	Appearance Impact	Aroma/Flavor Change	Final Score	Risk of Over-Tenderization
Control (Blind test)	—	—	—	—	3.9	None
Papain	0.05%	High—texture ↑ (1.3)	Moderate decline	Neutral	4.2	Moderate
Papain	0.1%	Moderate (1.0)	Noticeable decline	Neutral	3.3	High—over-tenderized
Bromelain	0.05%	High—texture ↑ (1.3)	Moderate decline	Slight aroma ↑	4.1	Mild
Bromelain	0.1%	—	Disqualified (visual degradation)	—	—	Very high
Ginger extract	10%	Highest—texture ↑ (1.4)	Minimal impact	None/pleasant	4.6	Not observed
Ginger extract	25%	—	Disqualified (visual degradation)	—	—	Very high

**Table 4:** SDS-PAGE results for the whole carcasses of garden snail—the estimated proportions of individual fractions in the protein of samples: untreated (“0”) or treated with buffer and enzymes.

			Papain		Bromelain		Ginger Extract	
Protein Fraction (kDa)	“0”	BUFFER	0.05%	0.10%	0.05%	0.10%	10%	25%
	Band %	Band %	Band %	Band %	Band %	Band %	Band %	Band %
>200	18.64	18.16	16.06	14.17	3.33	7.09	4.46	13.3
121–200	2.98	3.97	3.97	7.04	7.43	5.09	1.17	3.59
90–120	11.56	16.13	12.92	15.52	1.96	1.65	22.67	20.34
68–89	2.2	3.08	6.99	9.66	9.06	4.2	30.04	2.02
50–67	5.74	6.86	10.83	18.5	4.84	25.35	1.66	0
30–49	21.31	24.77	15.6	16.32	19.16	12.57	16.55	30.29
19–29	6.43	6.88	8	6	12.94	7.24	2.92	4.08
<19	31.14	20.17	25.66	12.77	41.28	36.81	20.55	26.36

**Table 5:** SDS-PAGE results for the feet of garden snail—the estimated proportions of individual fractions in the protein of samples: untreated (“0”) or treated with buffer and enzymes.

			Papain		Bromelain		Ginger Extract	
Protein Fraction (kDa)	“0”	BUFFER	0.05%	0.10%	0.05%	0.10%	10%	25%
	Band %	Band %	Band %	Band %	Band %	Band %	Band %	Band %
>200	23.07	19.56	24.53	22.12	8.55	8.54	19.74	8.89
121–200	4.22	3.25	5.04	5.26	4.53	1.9	5.91	2.9
90–120	16.03	16.36	14.45	16.84	15.58	10.1	17.11	21.56
68–89	1.39	2.33	4.7	4.5	2.02	2.7	3.01	2.96
50–67	6.85	7.19	4.4	4.08	12.18	19.04	5.23	2.37
30–49	25.39	29.54	21.06	20.3	20.05	24.61	24.3	32.03
19–29	6.26	5.93	7.14	8.53	8.42	10.77	12.01	15.47
<19	16.8	15.86	18.63	18.36	28.7	22.36	12.71	13.82

**Table 6:** Variants of enzymes concentrations for sample immersions.

Enzyme	Blind Test	1st Concentration Variant [%]	2nd Concentration Variant [%]
Papain	Citrate buffer solution (pH 6.0)	0.05	0.1
Bromelain		0.05	0.1
Ginger extract	water	10	25

**Table 7:** Guidelines for the sensory evaluation of tendered garden snail (*C. aspersum*) meat.

A Qualitative Feature	Weight Factor	Score Point				
		5	4	3	2	1
Appearance	0.20	Smooth, shiny surface, very well-preserved shape	Smooth surface, well-preserved shape	Matt surface, shape enabling recognition	Matte surface, poorly preserved shape	Completely not preserved shape, individual elements of the carcass visibly separated from each other
Colour	0.15	White or creamy	Creamy or yellow-cream	Beige	Grey or brown	Dark grey or dark brown
Aroma	0.15	Mild and characteristic	Very mild, poorly perceptible	Neutral	Indifferent, expressionless, with a perceptible mushroom/earthy smell	Intense fungal/earthy smell
Taste	0.20	Delicate, mild, specific taste	Mild, delicate, less palpable	Neutral, expressionless	Neutral, expressionless with a perceptible mushroom/earthy flavour	Intense fungal/earthy aftertaste
Texture	0.30	Soft and compact, flexible, allowing easy biting	Compact, slightly rubbery	Not very elastic, rubbery, hard, but it allows to bite	Rubbery, hard, difficult to chew	Very hard and rubbery, very difficult to chew/overly softened

## Data Availability

The original contributions presented in this study are included in the article. Further inquiries can be directed to the corresponding author.
